# The Ohio Medical School

**Published:** 1850-08

**Authors:** 


THE OHIO MEDICAL SCHOOL.

In addition to the election of Dr. John Bell, as Professor of the Theory and Practice of Medicine in the Medical College of Ohio, referred to in another place in this Journal, we find that Dr. T. O. Edwards has been elected Professor of Materia Medica and Therapeutics, and Medical Jurisprudence in the same college. Dr. Edwards distinguished himself in Congress by his efforts in regulating the importation of drugs, and we wish him success in his new vocation. B.

